# Clinical criteria and diagnostic assessment of fibromyalgia: position statement of the Italian Society of Neurology-Neuropathic Pain Study Group

**DOI:** 10.1007/s10072-023-06836-3

**Published:** 2023-05-24

**Authors:** G. Devigili, G. Di Stefano, V. Donadio, I. Frattale, E. Mantovani, M. Nolano, G. Occhipinti, V. Provitera, S. Quitadamo, S. Tamburin, A. Toscano, S. Tozza, A. Truini, M. Valeriani, M. de Tommaso

**Affiliations:** 1 UOC Neurologia IIRCCS Carlo Besta, Milan, Italy; 2 Department of Human Neuroscience, Sapienza University, Rome, Italy; 3 Clinica Neurologica Bellaria Hospital, Bologna, Italy; 4 Child Neurology and Psychiatric Unit, Tor Vergata University, Rome, Italy; 5 Neurosciences, Biomedicine and Movement Sciences Department, Verona University, Verona, Italy; 6 Skin Biopsy Laboratory, Department of Neurology, Instituti Clinici Scientifici Maugeri IRCCS, Telese Terme, Italy; 7 Department of Neurosciences, Reproductive Sciences and Odontostomatology, University Federico II of Naples, 80100 Naples, Italy; 8 UOC Neurologia E Malattie Neuromuscolari, AUO Martino Messina, Messina, Italy; 9 Developmental Neurology Unit, Ospedale Pediatrico Bambino Gesù, Rome, Italy; 10 EURO-ERN NMD, AOU Martino University of Messina, Messina, Italy; 11 Neurophysiopathology Unit, DiBraiN Department, Policlinico General Hospital, Bari Aldo Moro University, Piazza Giulio Cesare, 11, 70124 Bari, Italy

**Keywords:** Fibromyalgia, Diagnostic criteria, Small-fiber neuropathy, Laser-evoked potentials, Skin biopsy, Corneal confocal microscopy, Heart rate variability

## Abstract

**Background:**

The role of central and/or peripheral nervous system dysfunction is basically fundamental in fibromyalgia.

**Aim:**

The aim of this position statement on behalf of the Neuropathic Pain Study Group of the Italian Society of Neurology is to give practical guidelines for the clinical and instrumental assessment of fibromyalgia (FM) in the neurological clinical practice, taking into consideration recent studies.

**Methods:**

Criteria for study selection and consideration were original studies, case-controls design, use of standardized methodologies for clinical practice, and FM diagnosis with ACR criteria (2010, 2011, 2016).

**Results:**

ACR criteria were revised. For diagnostic procedure of small-fiber pathology, 47 studies were totally considered.

Recent diagnostic criteria should be applied (ACR, 2016). A rheumatologic visit seems mandatory. The involvement of small fibers should request at least 2 among HRV + SSR and/or laser-evoked responses and/or skin biopsy and/or corneal confocal microscopy, eventually followed by monitoring of metabolic and/or immunological/ and or/paraneoplastic basis, to be repeated at 1-year follow-up.

**Conclusions:**

The correct diagnostic approach to FM could promote the exclusion of the known causes of small-fiber impairment. The research toward common genetic factors would be useful to promote a more specific therapeutic approach.

**Supplementary Information:**

The online version contains supplementary material available at 10.1007/s10072-023-06836-3.

## Introduction

Fibromyalgia (FM) is a complex and common syndrome, whose main clinical element is represented by chronic non-specific pain associated with numerous and various other symptoms, linked to the involvement of the nervous system such as fatigue, insomnia, and emotional distress. Pain in FM is most typically classified as nociplastic; the terminological definition of nociplastic pain was formulated by the IASP in 2017 and defined as “pain that arises from altered nociception despite no clear evidence of actual or threatened tissue damage causing the activation of peripheral nociceptors or evidence for disease or lesion of the somatosensory system causing the pain” [1]. The need to classify pain in a new third category originates from the observation of a more elusive subtype of pain with mechanisms that are not yet fully understood, compared to the two classic typologies of pain, nociceptive and neuropathic pain, caused respectively by non-neural tissue damage, producing an activation of nociceptors with a normal functioning of the somatosensory nervous system, and by damage to the somatosensory system [2]. Nonetheless, it is evident that FM patients may experience overlap between the three different types of pain, nociceptive and neuropathic in addition to nociplastic pain [2], even in the presence of normal functioning of the somatosensory nervous system [3]. A more complete understanding of the pathophysiological mechanisms of pain may in the future lead to an overcoming of the current pain classification.

Impaired pain processing, consisting of central pain amplification or decreased pain modulation along the descending pathway, or a combination of these two types of dysfunctions, is the most distinctive element in FM [2]. However, it is currently not clear whether the detectable alterations have a primary or secondary origin at the level of the central or the peripheral nervous system, indeed such alterations being identifiable at both levels. At the same time, a predominantly non-length-dependent neuropathy, of a non-developmental type, frequently accompanies patients with FM [4].

Considering that FM is frequently comorbid with autoimmune pathologies and since in patients with FM there are measurable altered cytokine levels, it has been hypothesized that this syndrome may be due to autoimmune processes, as demonstrated in mice by transfer of purified IgG from individuals with FM [5].

The complex and only partially known pathophysiological basis in addition to diagnostic criteria based only on subjective symptoms in the absence of specific clinical signs and recognized biomarkers means that FM is underdiagnosed and undertreated. For these reasons, an aura of stigma and the burden of inadequate medical and social support still weigh on the affected patients.

The clinical features included in the diagnostic criteria are largely attributable to central and/or peripheral nervous system dysfunction; so far, the role of the neurologist is of evident utility in the diagnosis and clinical management of FM.

The aim of this position statement on behalf of the Neuropathic Pain Study Group of the Italian Society of Neurology (SIN) is to give practical guidelines for the clinical and instrumental assessment of FM in the neurological clinical practice, taking into consideration recent studies.

## Methods

This is a position statement on diagnostic assessment of fibromyalgia, based on a not structured review.

### Eligibility criteria and search strategy

For clinical criteria, we took into consideration the American College of Rheumatology criteria for adult and childhood fibromyalgia.

**Fig. 1:**
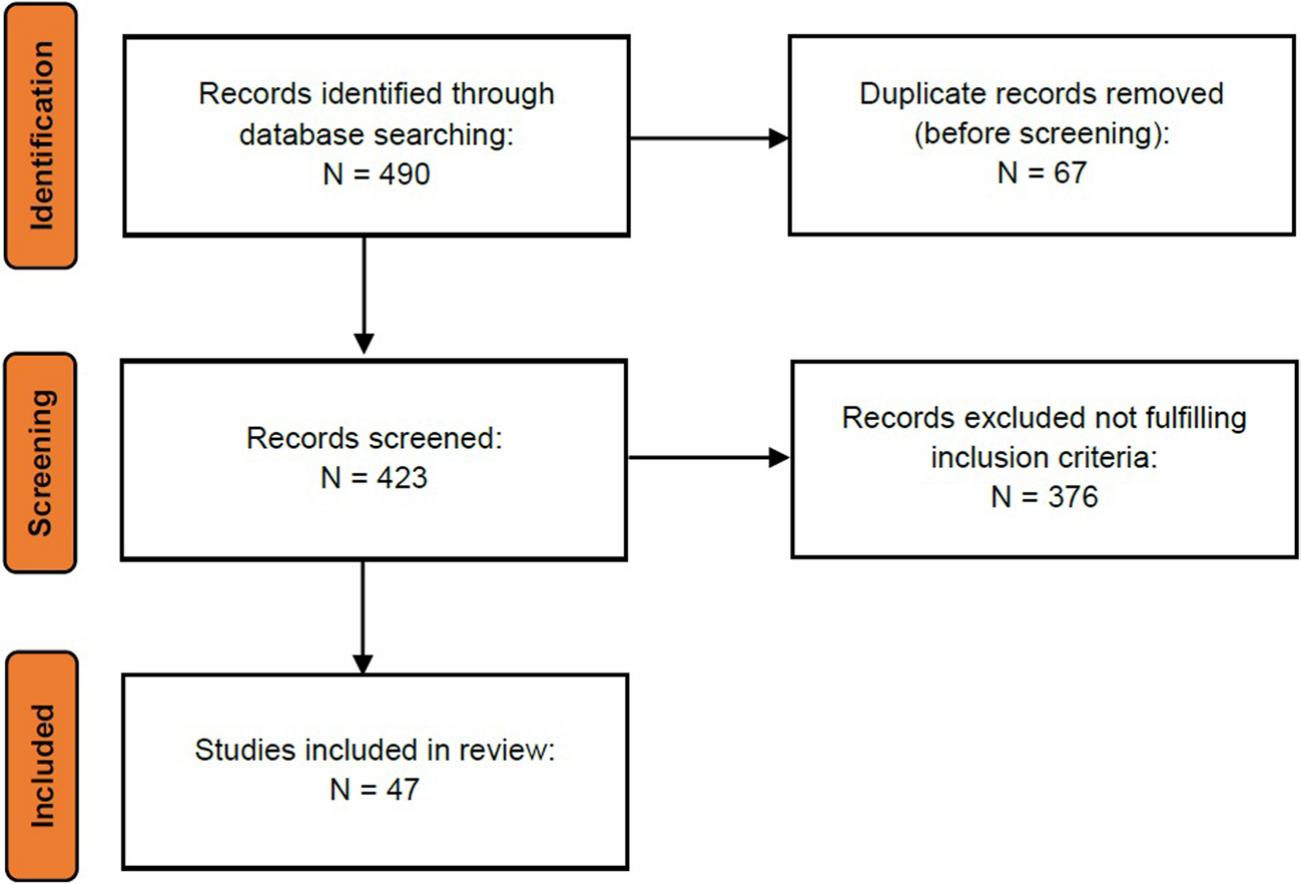
Flow chart showing the total number of identified, screened, included, and excluded records

For instrumental assessment, studies on neurophysiological, psychophysical, autonomic, and other instrumental techniques applied to clinical assessment of FM, with special regard to sensory and autonomic neuropathy, were searched on PubMed. The search strings are reported in Table 1. The search was not filtered for study type. Two authors independently assessed studies and any disagreement was planned to be solved by consensus with a third author. Main original data were thus revised. Criteria for study selection and consideration were: original studies, case-controls design, use of standardized methodologies for clinical practice, FM diagnosis with ACR criteria (2010, 2011, 2016). Reviews and case reports were not considered. Figure 1 reports the flow chart showing the total number of identified, screened, included, and excluded records. We thus took into consideration studies in the last 10 years, and summarized publications within 5 years (2018–2022) in tables.

**Table 1:** Total number of studies selected with the key words. Main studies are described in detail in the next paragraphs. When a study included more than one technique, it was reported only as the first occurrence

Instrumental technique	Search string	Retrieved and selected records
Nerve conduction study	(fibromyalgia) AND (nerve conduction studies OR electroneurography)	Identified 26 Screened 26 Included 5 [6-11]
Laser–nociceptive evoked potential or pain-related evoked potentials	(fibromyalgia) AND (laser-evoked potentials or LEP or” nociceptive evoked potentials” or “pain related evoked potentials”)	Identified 32 Screened 32 Included 9 [3, 12-19]
Autonomic assessment	(fibromyalgia) AND ("sympathetic dysfunction" OR "autonomic assessment" OR "heart rate variability" OR "Quantitative Sudomotor Axon Reflex Test" OR “QSART” OR Valsalva OR Tilt test OR Sudoscan OR "sympathetic skin response" OR "SSR" NOT SSR [journal])	Identified 147 Screened 147 Included 10 [20-29]
Skin biopsy	(fibromyalgia) AND ("skin biopsy" OR small-fiber neuropathy OR "intraepidermal nerve fiber density" OR IENFD)	Identified 99 Screened 99 Included 11 [17, 30-39]
Corneal confocal microscopy	(fibromyalgia) AND ("corneal confocal microscopy" OR CCM OR "corneal innervation")	Identified 14 Screened 14 Included 5 [40-44]
Microneurography	(fibromyalgia) AND (microneurography)	Identified 5
		Screened 5
Included 2 [45, 46]		
Quantitative sensory testing	(fibromyalgia) AND (quantitative sensory testing OR psychophysics OR "QST" NOT QST [journal])	Identified 167 Screened 167 Included 5 [46-50]

Search strings and retrieved records are reported separately for each considered instrumental technique in Table 1.

## Diagnostic criteria

### Fibromyalgia in adult age

The first studies on FM date back to sixteenth century observations, which saw slow progress toward the formulation of recognized diagnostic criteria. The term FM was coined only in 1976, from the union of the words “fibro” and “mio,” to highlight the involvement of the fibrous and muscular tissue and the word “algia,” indicating pain as an essential element for the diagnosis. The first diagnostic criteria were formulated by a scientific society only in 1990, thanks to the lack of prejudices and the farsightedness of the College of Rheumatology (ACR), which, despite the absence of clinical tests to objectify the pathology, believed it could not ignore this condition. The 1990 diagnostic criteria emphasized the role of tender points, tenderness of at least 11 of 18 specific body sites, and the role of diffuse pain defined as pain persisting for at least 3 months in both supra-pelvic and subpelvic regions in addition to axial pain [51].

A strong limitation of these criteria was represented by the fact that at least 25% of patients affected by FM do not fully satisfy the ACR criteria proposed, since in addition to the subjectivity of the response to the pressure exerted, as well as the difficulty for clinicians to apply 4 kg of strength, it has been widely experienced that acupressure tenderness in male subjects appears less evident than in female subjects, leaving many male FM patients undiagnosed. Despite the evident limitations of the first diagnostic criteria, they are considered instrumental in the recognition of the pathology and in its inclusion by the consensus group of the Declaration of Copenhagen (CD) among non-articular rheumatism in the International Classification of Disease of the World Health Organization Healthcare with the ICD code M 79.0 (ICD 10th revision).

Hence, in 2010, the ACR also published the first revision of the criteria, in which the search for the positivity of tender points has seen its diagnostic role disappear, replaced by a greater weight given to subjective symptoms [52]. Subsequently, in 2011, the same authors proposed a modified version of the ACR criteria to allow diagnosis through a self-assessment based on six somatic symptoms considered predominant (non-refreshing sleep, fatigue, cognitive deficits, headache, depression, and abdominal pain) among a list of 41 symptoms proposed in the Symptom Severity Score (SS) of the 2010 ACR criteria to be reserved for research purposes only and to introduce a quantitative measure of disease severity, the Fibromyalgia Severity Scale (FS score, range from 0 to 31) resulting from the sum of the widespread pain index (WPI) score and the modified SS score [53]. The last revision of the ACR criteria dates back to 2016 and changes the definition of widespread pain, defined as present in at least four of five regions (excluding the jaw, chest, and abdomen); specifies somatic symptoms such as headache, pain, or cramps abdominal pain and depression; adds the FS scale, and introduces the criterion according to which the diagnosis of FM should be done regardless of other diagnoses and that the diagnosis of FM must not lead to the exclusion of the presence of other relevant concomitant pathologies [54].

### Fibromyalgia in children

Although fibromyalgia is highly prevalent in adulthood, it can be present also in the developmental age. In children and adolescents, its prevalence is poorly studied and varies between 7 and 15% of new visits to pediatric rheumatology clinics [55]. Girls/boys ratio is 4:1 and age at diagnosis ranges from 13 to 15 years, although diagnoses at younger ages are reported [56].

Diagnostic criteria for juvenile fibromyalgia (JFM) were first described in 1985 by Yunus and Masi [57] and subsequently revised in 2010 [58], with peculiarities as compared to the criteria for adulthood. Laboratory and diagnostic tests are often negative [56]. Clinically, JFM is characterized by chronic and widespread pain, which can be spontaneous or evoked by painful or non-painful stimuli. It is associated with fatigue and highly disabling symptoms [59]: headache in two-thirds of cases, abdominal pain [60], sleep disorders, such as periodic limb movement [61], and psychiatric disorders, such as depression and anxiety [62, 63].

Growing pains can be considered precursors of JFM [64].

## Diagnostic procedures

In the following paragraph, we summarize the main studies on technical procedures of small-fiber function and autonomic system assessment that fulfilled our inclusion criteria (Fig. 1). *Nerve conduction studies-NCS- and electromyography-EMG- *Apart from few studies in favor of electroneurography signs compatible with sensory neuropathy [6, 7], nerve conduction study and electromyography are generally within normal limits in FM patients [8-11]. Lawson et al. found a mild sural and medial plantar (MP) response amplitude reduction in those subjects reporting symptoms of neuropathic pain, distal small-fiber neuropathy, and markers of metabolic syndrome [7]. The lack of a previous neurological assessment [6] or the presence of signs of neuropathic pain [7] could suggest cautiousness in interpreting abnormal NCV findings, but their routine execution in the presence of clinical signs of polyneuropathies or pain of neuropathic origin seems strongly recommended.

### Laser and nociceptive evoked potentials

Ten studies tested pain-related evoked potentials in patients with FM. Four studies showed increased amplitude of laser-evoked potentials (LEPs) and reduced habituation in patients with FM, supporting an abnormal central elaboration of pain and pain matrix hyper excitability [12-15]. Accordingly, Aδ LEP amplitude, conditioned by a preceding C-fiber LEP, was significantly higher in patients with FM than in healthy controls, supporting the hypothesis of pain matrix hyper excitability [16].

Three studies used pain-related evoked potentials (PREPs) to detect small-fiber pathology in FM with contrasting findings [3, 17, 18]. In a case-control study involving 25 patients with FM, Üçeyler and colleagues reported reduced amplitudes of pain-related evoked potentials elicited by surface concentric electrodes in patients with FM compared to control subjects, supporting abnormalities of small fibers [17]. Two studies, using LEPs, did not confirm Aδ fiber function abnormalities, suggesting that small-fiber pathology negligibly affects somatosensory system function in FM and is not a significant contributor to the pathophysiology of this condition [3, 18]. In the study of Fasolino and colleagues in 57 patients with FM, LEP parameters fell within normative ranges and did not differ in patients with or without small-fiber pathology, detected through skin biopsy [3]. In the study of Van Assche and colleagues, none of the 92 patients enrolled showed signs of loss of function of the nociceptive responses evoked by Aδ fiber activations [18].

Two recent studies assessing multichannel LEPs in patients with FM and small-fiber impairment showed abnormal N2P2 habituation index, more marked in patients with reduced IENFD, and reduced amplitude of the P2 component, not coherent with the site of denervation [11, 19] (Table 2). In summary, laser-evoked potentials reflect abnormal pain processing at both peripheral and central levels in FM, with amplitude amplification occurring in the presence of peripheral denervation. Nevertheless, clear amplitude loss or even the absence of cortical components should be considered as a sign of severe Aδ fiber impairment (Table 2).

**Table 2:** Studies about laser evoked potentials (LEPs) in fibromyalgia (FM) and healthy controls (HC) published within the last 5 years. In bracket, disease duration in years is reported, when available

Authors	Methods	Subjects	Results	Significance
De Tommaso et al. (2017)	CO_2_ laser stimulation	50 FM (7.2 ± 6.21 y) and 30 HC	Reduced habituation of vertex LEPs	Finding supporting abnormal central elaboration of pain
Fasolino et al. (2020)	Nd:YAP laser stimulation	57 FM (7 ± 8.5 y)	Normal LEP parameters in patients with and without small-fiber pathology	Finding suggesting that small-fiber pathology has a negligible impact on somatosensory system function in fibromyalgia
Van Assche et al. (2020)	CO_2_ laser stimulation	92 FM 39 HC	Normal LEP parameters	Finding suggesting that small-fiber neuropathy is not a significant contributor to the pathophysiology of fibromyalgia
Vecchio et al. (2020)	CO_2_ laser stimulation	81 FM (10.69 ± 8.16 y)	Abnormal habituation index and correlation with reduced IENFD at the thigh	Finding suggesting central impairment of pain processing and association with mild proximal small-fiber pathology
Vecchio et al. (2022)	CO_2_ laser stimulation	37 FM (10.27 ± 7.17 y) (22 patients with proximal denervation, 18 with normal skin biopsy, and 7 with proximal and distal IENFD reduction	Reduced amplitude of the P2 component, not coherent with the site of denervation Decreased habituation of P2 prevailing in patients with reduced IENFD	Finding suggesting that LEP abnormalities are not the expression of small-fiber impairment in fibromyalgia

Sympathetic skin response — SSR — and autonomic assessment (heart rate variability-HRV)

Patients with FM, beside pain, often present a constellation of complaints, like persistent fatigue, gastrointestinal dysmotility, dizziness, and syncope that can be expression of a dysfunction of the autonomic nervous system (ANS). Also, in patients with a history of vasovagal syncope, a higher prevalence of FM compared to the general population has been reported [20]. Different hypotheses have been formulated to explain autonomic disturbances in fibromyalgia, including abnormal hypothalamic–pituitary axis functioning and autonomic small-fiber impairment. Many studies, most of them based on the analysis of heart rate variability (HRV) at rest or following physical challenges, have found ANS dysfunctions in FM [21-28]. The idea has prevailed that FM is characterized by an altered sympatho-vagal balance with an increase in sympathetic and decrease in parasympathetic activity. Few studies assessed sudomotor function in FM.

Sympathetic skin response amplitude in patients is not generally different from controls [15, 29]. However, it correlates with disease severity as assessed by clinical questionnaires [29]. Conversely, SSR latency has been found to be significantly longer in FM compared to controls and the response totally absent in a percent varying from 15 to 18% of patients [15]. Moreover, using electrochemical skin conductance analysis, sudomotor function has been found to be significantly impaired in patients with FM [29]. Autonomic symptoms are more severe in women affected by FM who also complain of more intense pain [27]. Moreover, a significant correlation has been found between the prevalence of autonomic symptoms and the impact of FM on general health status [25].

In conclusion, the data present in literature are strongly oriented toward an association between FM and dysautonomia that further deteriorates patients’ quality of life, so the evaluation of autonomic involvement could offer information on the severity of the disease (Table 3).

**Table 3:** Studies about autonomic system assessment with hearth rate variability and sympathetic skin response published within the last 5 years. In bracket, disease duration is reported, when available. FM, fibromyalgia; HC, healthy controls

Authors	Methods	Subjects	Results	Significance
Reyes-Manzano et al. (2018) [23]	Cardiovascular reflexes	30 FM patients 30 HC (not reported)	Reduction of multifractality of RR fluctuations	Dysfunction of ANS
Pickering et al. (2020) [29]	Electrochemical skin conductance	50 FM patients 50 HC (12 ± 3 years)	Reduced ESC values in FM patients	Impaired sudomotor function
Hazra et al. (2020) [24]	Cardiovascular reflexes	50 FM patients 50 HC (42.8 ± 37.1 months)	Normal parasympathetic activity Sympathetic hyperactivity in FM	Altered sympatho-vagal balance
Singh et al. (2020) [25]	Cardiovascular reflexes	30 FM patients 30 HC	Autonomic dysfunction in 40% of FM patients	Dysfunction of ANS
Schamne et al. (2021) [26]	Cardiovascular reflexes	23 FM patients 17 HC (7.7 ± 7.1 years)	Lower HR max, HR reserve, and chronotropic reserve in FM during exercise	Impaired parasympathetic activity
Rost et al. (2021) [27]	Cardiovascular reflexes	46 FM patients 46 HC (189.9 ± 117.8 months)	Reduced HRV in FM patients	Impaired parasympathetic activity
Sochodolak et al. (2022) [28]	Cardiovascular reflexes	35 FM patients 17 HC (moderate FM 8.50 ± 6.07 years; severe FM 8.57 ± 6.68 years)	Reduced HRV in FM patients	Impaired parasympathetic activity
Papadopoulou et al. (2022)	SSR	21 FM patients 28 HC	SSR latency correlated to pain intensity	Association between pain and autonomic impairment

### Skin biopsy

In the last decade, several studies showed evidence of small-fiber pathology in a variable proportion of patients with FM, with lower intraepidermal nerve fiber density (IENFD) compared with healthy subjects [3, 10, 11, 14, 17, 30-32]. A similar finding has also been reported in JFM [33]. Heterogeneity between studies and, often, small population size prevented conclusive findings. Skin denervation was mostly reported as not length-dependent pattern [3, 17, 34]; however, in a subset of patients, a length-dependent distribution has been described [10, 11], leading to a challenging distinction from the small-fiber neuropathy. The presence of a more severe pain phenotype and anxiety associated with generalized denervation has been also reported [4, 10]. Myelinated dermal fibers have been found to be usually spared [14, 17], although a loss of Meissner corpuscles at fingertips has been observed [14]. In addition to nerve loss, morphological changes such as lower density of regenerating nerve fiber positive to growth-associated protein (GAP) 43 [17] and a reduction of diameter in dermal nerve fibers have also been reported [8]. The observation of increased peptidergic innervation of cutaneous arteriole-venues shunts in the glabrous skin of patients with fibromyalgia [34] suggested a neurogenic microvascular dysfunction that may imply the coexistence of peripheral mechanisms underlying pain [17, 35-38]. Along with cutaneous denervation at proximal leg, an aberrant cutaneous and systemic miRNA expression pattern was found in patients with fibromyalgia [34]. Finally, the finding of reduced mitochondrial chain activities and bioenergetics levels and increased levels of oxidative stress in the skin biopsies of 20 women with FM compared to controls [39] suggests a role of oxidative stress, mitochondrial dysfunction, and inflammation in the pathophysiology of pain in fibromyalgia. (Table 4). Skin biopsy is the elective test for small-fiber pathology. Its evaluation in patients with FM is suggested to ensure small-fiber involvement.

**Table 4:** Studies on skin biopsy in fibromyalgia (FM) patients published within the last 5 years. In bracket, disease duration is reported, when available. FM, fibromyalgia; HC, healthy controls

Authors	Methods	Subjects	Results	Significance
Lawson et al. (2018) [31]	Skin biopsy NCS	155 FM	Reduced IENFD 43 patients (28%) at distal site 19 (12%) at proximal site. Sensory amplitudes correlation with distal IENFD	Identification of subset of FM with small fiber early or mild sensory neuropathy and metabolic disease
Evdokimov et al. (2019) [10]	Skin biopsy, corneal confocal microscopy, microneurography, quantitative sensory nociceptive evoked potentials	117 FM (mean 12 years, range 0.8–56) 54 HC 11 women with major depressive disorder and chronic widespread pain (mean 5 years, range 1–44)	4 distinct FM subgroups: normal skin innervation (FM 37%, controls: 82%), reduced distal IENFD (FM: 17%, controls: 13%), reduced proximal IENFD (FM: 31%, controls: 2%) proximal and distal reduction in IENFD (FM: 15%, controls: 2%)	Higher pain intensity and anxiety in FM patients with generalized IENFD reduction
Evdokimov et al. (2020) [38]	Skin biopsy Epidermal and dermal nerve quantification	86 FM (mean 4 years, range 0.5–35) 35 HC	IENF reduction in 38 FM with reduced nerve length in close proximity to blood vessels	Altered peripheral circulation in FMS patients
Evdokimov et Al. (2020) [36]	Skin biopsy (IENFD and primary fibroblast and keratinocyte cell cultures). PCR (gene expression of selected pro-and anti-inflammatory cytokines, nociception-associated ion channels, and axon guidance cues)	128 FM 26 controls	In FM, higher expression of TGF-ß1 gene, HCN2, EFNA4, and EPHA4 in fibro-blasts and IL-10 gene in Keratinocytes	Membrane bound and soluble pain mediators and axon pathfinders as contributors to cutaneous nociception in FM
Fasolino et al. (2020) [37]	Skin biopsy QST LEP	57 FM (mean 5 years, range 2–9.5)	18 patients with non-length-dependent loss of IENFD but without abnormalities in QST and LEP	Negligible impact of small-fiber pathology on somatosensory system function in fibromyalgia
Vecchio et al. (2021) [11]	Skin biopsy LEP NCS	81 FM (mean 10.7 years, range 1–20)	59 FM proximal IENF loss; 10 with IENFD distal and proximal loss No association among IENFD, LEP, clinical features, and comorbidities. Normal NCV	Central impairment of pain processing and mild proximal small-fiber pathology in the most of FM
Bonepart et al. (2021) [32]	Skin biopsy	15 patients with juvenile fibromyalgia (range 21–26 months) 23 controls	Reduced IENFD in 8 out of 15 FM patients	IENF reduction is present in adolescents with FM, with similar prevalence as adults
Quitadamo et al. (2022) [4]	Skin biopsy	62 FM (mean ± SD 9.69 ± 7.21 years) clinical and skin biopsy 18-month follow-up	Stable IENFD at follow-up. Length-dependent small-fiber reduction predicted more severe disability and reduced response to drugs and physical exercise	Small-fiber impairment seems stable in medium term in FM, but it can predict FM outcome

#### CCM

Considering the elective innervation of the cornea by C fibers, corneal confocal bio-microscopy is a reliable method to assess small nerve fiber pathology. While the study by Oudejans clearly reported diagnostic cut-off values and relied on widely agreed parameters including nerve fiber length, nerve fiber density, and branching, the study by Ramirez investigated different parameters (stromal nerve thickness and corneal sub-basal plexus nerve density) and did not provide diagnostic cut-off values [40, 41]. More recent studies confirmed utility of corneal confocal microscopy in FM with abnormalities in most of the patients, frequently in association with severity of clinical expression [10, 42]. Changes in corneal innervation and Langerhans cells were detected in FM patients and those with small-fiber neuropathy [43]. To summarize, corneal confocal microscopy confirmed the presence of small-fiber impairment in FM, although the clinical picture did not resemble neuropathic pain, especially in patients with severe anxiety and depression [44]. Its evaluation in FM patients could reliably confirm impairment of small afferents (Table 5).

**Table 5:** Studies on corneal confocal microscopy (CCM) in fibromyalgia (FM) patients published within the last 5 years. In bracket, disease duration is reported, when available. FM, fibromyalgia; HC, healthy controls

Authors	Methods	Subjects	Results	Significance
Erkan Turan et al. (2018) [42]	(CCM) Nerve fiber density and tortuosity	34 FM 42 HC (6.9 + 5.1 years)	Lower epithelial cell density in FM, correlated with increased widespread pain index (WPI)	Utility of corneal confocal microscopy in clinical assessment of FM
Van de Donk et al. (2018) [43]	CCM fiber length and density, double-blind placebo controlled trial with tapentadol	34 FM (tapentadol group 5.4 + 4.9, placebo 4.8 + 3.8 years) Laboratory reference	13 FM patients with corneal fiber density and length abnormalities Prevalence in tapentadol not responders	CCM could predict therapeutic response to tapentadol
Evdokimov et al. (2019) [10]	CCM fiber density, branching density, and nerve fiber length. (microneurography, QST, PREPs, skin biopsy)	117 FM (mean 12 years, range 0.8–56) Laboratory reference 54 HC 11 patients with depression and widespread pain	Corneal nerve fiber density reduced in FM, not in patients with depression. Correlation with severity of intrapidermal nerve density reduction	Corneal denervation parallels skin denervation and is associated with severe FM
Klitsch et al. (2020) [44]	CCM Langerhans cells-dendritic and non-dendritic, corneal nerve fiber density, length, and branch density	134 FM (12; 0.75–56 years) 41 SFN (4; 0–20 years) 60 HC	Fewer dendritic Langerhans cells, nerve fiber length and density in FMS and SFN, branch density reduced in SFN patients	Changes in corneal innervation and Langerhans cell distribution in FMS and SFN
Ramirez et al. (2020) [41]	CCM fiber density, length, and branch density (correlation with neuropathic pain scores: assessment of anxiety and depression)	28 FM (4.71 + 6.2 years)	Strong negative correlation between neuropathic pain scored and corneal nerve density in the subgroup of FM women without severe anxiety or depression (*n* = 13)	Severe anxiety or depression distorts fibromyalgia symptoms

### Microneurography

Microneurography represents an ideal technique to explore the peripheral nociceptor system and to record intraneural single C-fiber action potentials from nociceptors. Multiple C-fibers can be recorded simultaneously for hours [65], thereby allowing the assessment of individual firing behavior to various external stimuli.

The application of microneurography in FM is unfortunately limited. Using this technique, C-nociceptors were recorded in female FM patients [45]. The results were compared with those collected in female patients with small-fiber neuropathy and controls. The majority of FM patients showed abnormal firing of C-nociceptors. Many silent nociceptors exhibit hyper excitability resembling that in small-fiber neuropathy, but high activity—dependent slowing of conduction velocity is more common in FM patients, and may constitute a hallmark. The main conclusion of this study was that abnormal peripheral C-nociceptor ongoing activity and increased mechanical sensitivity could contribute to the pain and tenderness referred by FM patients [45]. Essentially, the same results were found in a second paper from the same group [10] showing that FM patients showed hyper excitability and sensitization of mechanoresponsive (1A fibers) and mechano-insensitive (1B) nociceptors. If confirmed this is an important contribution as it may suggest that peripheral nociceptors are functionally abnormal in FM in agreement with the morphological abnormalities disclosed by skin biopsy. An important limitation of these papers is the lack of correlation with morphological analysis of peripheral nociceptors that prevent to correlate the functional abnormalities highlighted by microneurography with the morphological abnormalities disclosed by skin biopsy. Presently, microneurography seems a promising tool to evaluate nociceptors abnormalities, but the procedure is of limited diffusion in clinical practice with few application in patients with FM.

#### QST

Quantitative sensory testing (QST), a standardized method used to assess somatosensory system function, if a comprehensive protocol is applied, allows to detect sensory and pain profiles, and to hypothesize the underlying mechanisms (i.e., peripheral versus central nervous involvement, focal versus generalized sensory dysfunction) [46].

In fibromyalgia, QST findings showed a certain heterogeneity in the sensory threshold abnormalities. However, a common result between different studies is the presence of hypersensitivity or gain of function to a broad of standardized sensory stimuli [8, 10, 17, 33, 47, 48]. In particular, increased mechanical pain sensitivity (MPS) and reduced pressure pain thresholds (PPT) are commonly reported [3, 17], that suggest prevalent mechanism of central sensitization [46]. Moreover, a high rate of aftersensation after mechanical stimuli is also described [47] while, less commonly, mechanical dynamic allodynia is reported [3, 40, 47]. Additionally, in a subset of patients, also, mechanical detection thresholds (MDT) can be increased, that, unrelated to other evidence of large nerve fiber impairment, probably is supposed to be due to impaired C-tactile afferents in the context of a small-fiber pathology [10].

Regarding thermal stimuli, several studies described cold pain hyperalgesia [10], and increased cold and warm detection thresholds [3, 8, 10, 17], although other authors reported normal thermal thresholds [48].

Compared with small-fiber neuropathy, patients with FM with or without epidermal denervation have different sensory phenotypes [48], suggesting that despite the coexistence of skin denervation and small-fiber pathology, SFN and FM have different mechanisms underlying pain [3, 48, 49]

Finally, the application of dynamic protocols of temporal summation and conditioned pain modulation (CPM) showed significant dysfunction of the diffuse noxious inhibitory control (DNIC) [29, 50], rising the hypothesis of altered endogenous pain mechanisms, compared with healthy controls. (Table 6)

**Table 6:** Studies on quantitative sensory testing application in fibromyalgia published within the last 5 years. In bracket, disease duration is reported, when available. FM, fibromyalgia; HC, healthy controls; SFN, small-fiber neuropathy

Authors	Methods	Subjects	Results	Significance
Evdokimov et al. (2019) [10]	QST battery (13 parameters) (skin biopsy, CCM microneurography, PREP)	117 FM (mean 12 years, range 0.8–56) 34 HC	Different subgroups; worse hyperalgesia in generalized IENFD reduction	Existence of different clinical features, more severity related to small-fiber pathology
Wodehouse et al. (2018) [50]	QST (only mechanical pressure stimuli) and CPM using tonic and phasic mechanical pressure pain	25 FM 14 completed follow-up	Pressure pain sensitivity Diffuse noxious inhibitory controls (DNIC) less efficient Both improve after treatment 12 weeks of pregabalin treatment	Modulation of pressure pain and DNIC efficacy after pharmacological treatment (pregabalin)
Pickering et al. (2019) [29]	QST (cold and warm thresholds) CPM to assess DNICs ESC	(12 + 3 years)	In fibromyalgia, DNIC is less functional, cold and warm detection threshold higher, thermal pain thresholds reduced	Altered endogenous pain mechanisms
Fasolino et al. (2020) [3]	QST battery 10 parameters (skin biopsy, voltage-gated channel genotyping)	57 FM (mean 5 years, range 2–9.5)	Increased mechanical pain sensitivity, no difference between pts with or without skin denervation	Central sensitization as a major mechanism Small-fiber pathology does not affect somatosensory system
Rehm et al. (2021) [49]	QST battery (13parameters)	87 FM (12.95 + 9.03)	Different somatosensory pattern Hypersensitivity for noxious mechanical and thermal stimuli. Pinprick hyperalgesia or dynamic mechanical allodynia	Central sensitization as a major pathophysiological mechanism
Berwick et al. (2022) [47]	QST mechanical pain thresholds; pressure pain thresholds; brushstroke pain	44 FM (10.6; 8.912.3 years)16 HC	Pain after pressure aftersensation to mechanical not painful stimuli (77%), and dynamic mechanical allodynia in 10%	Evidence of central sensitization
Leone et al. (2022) [48]	QST 10 parameters	64 FM 20 SFN (5.1; 0.4–20 years), 44 without SFN (6.4; 0.5–14 years) 30 pure SFN	Different sensory phenotype between FM (with or without SFN) and SFN. In FM, presence of hyperalgesia phenotypes (both thermal and mechanical) or normal profile, compared with SFN (sensory loss and mechanical hyperalgesia)	The study supports the present of a complex mechanisms underlying pain Small-fiber pathology does not significantly affect somatosensory system

In conclusion, considering the consistent methodological heterogeneity between studies, future research needs to investigate with a multimodal approach, the potential role of QST-based algorithm in stratification of FM patients for clinical and research purposes.

### Position statement

**Fig. 2:**
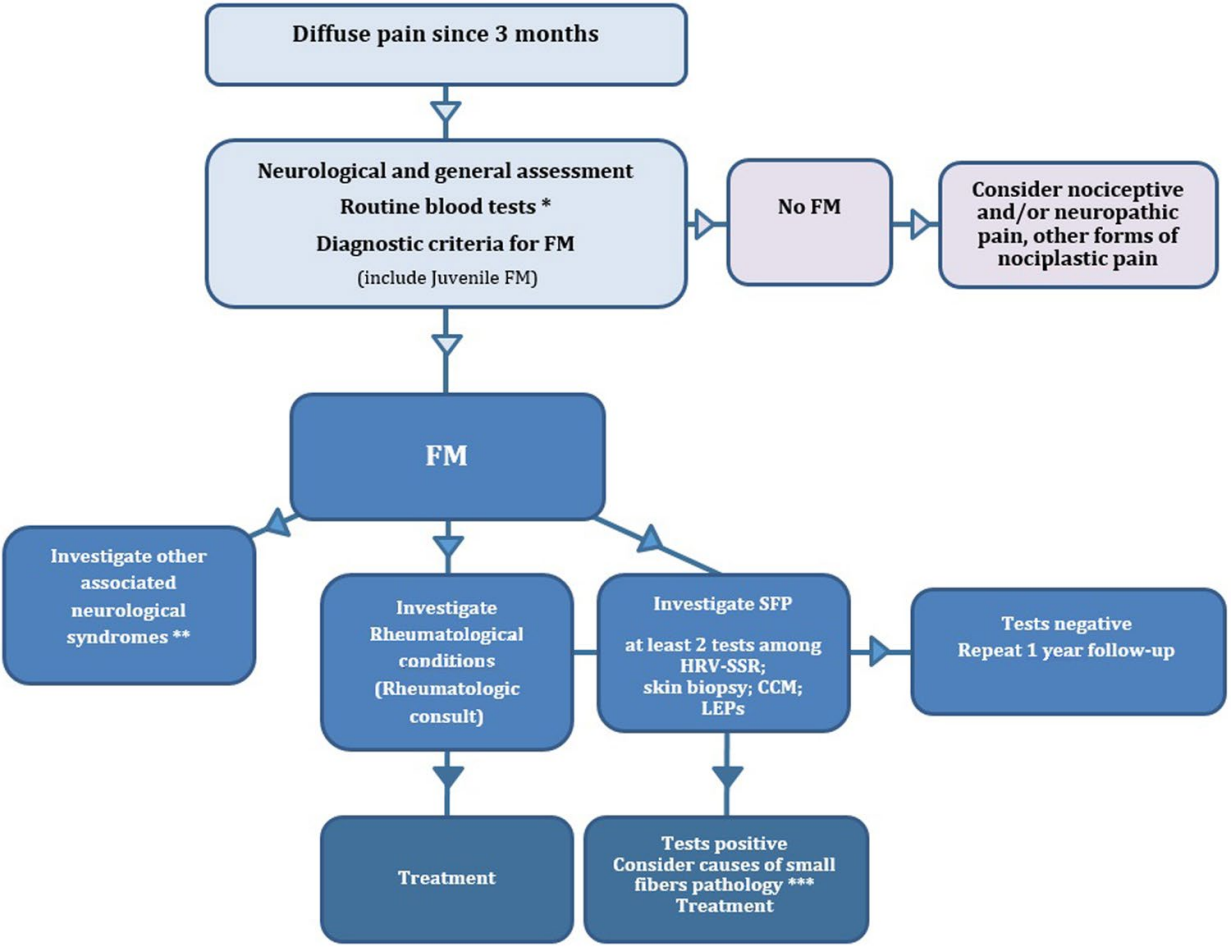
Flow chart reporting the proposal simplified diagnostic assessment for patients with FM. *Glucose dysmetabolism assessment: renal, thyroid, and liver function tests; B12 vitamin and folate blood levels; erythrocyte sedimentation rate; blood cell count. **For associated neurological conditions, consider the supplementary section. ***For tests useful to consider causes of small-fiber neuropathy, consider Gemignani et al. [66]

Neurological management of FM patient-clinical criteria are as follows:

1. Patients with generalized pain should be taken into consideration for FM diagnosis.
2. Recent diagnostic criteria should be applied (ACR, 2016). Diagnosis of FM is based on the sum of WPI (WPI) > 7 and symptom severity scale (SSS) score > 5 OR WPI of 4–6 and SSS score > 9. Severity of FM is also based on the sum between WPI and SSS scores. For fatigue, waking unrefreshed, and cognitive symptoms, the questionnaire should be followed. For headache comorbidity, International Headache Society criteria [66] should be applied. For depression, neurologist should consider the presence of depressive symptoms, using clinical impression, sustained by clinical criteria (DSM V). For abdominal pain, the description of such symptom since at least 3 months should be considered. For juvenile age, the same criteria should be applied, with the recommendation to be administered by a clinician or health care professional. In addition, the WPI would consider the last 3 months, instead of the last week [58].
3. A careful investigation of personal and familiar history of neurological conditions, as well as a detailed objective examination of neurological deficit, with particular regard to multimodal sensations, including tactile, vibration, nociceptive, and thermal ones, seems mandatory in the clinical approach of patients with suspected FM. The huge expression of central sensitization phenomena could contribute to hide myopathies and central and peripheral neurogenic pathologies. In the new classification, other conditions leading to pain do not exclude FM, so the definition of associated syndromes outside those included in the classification should complete the diagnosis. The clinical neurological examination would contribute to define deficit in central and peripheral nervous system, and to proceed with the adequate diagnostic measures. In the supplementary section, the neurological diseases with diffuse pain with possible association with FM are described (Supplementary section).
4. A rheumatologic visit should also be requested, if lacking, to clarify frequent comorbid conditions
5. In the case of FM diagnosis, the involvement of small somatic and autonomic fibers, frequently not associated to objective signs of sensitive and autonomic dysfunctions, would request the diagnostic procedures detailed in the above reported paragraph. At least two among HRV + SSR and/or laser-evoked responses and/or skin biopsy and/or corneal confocal microscopy should be suggested. Microneurography is rarely available. The QST could also be suggested, although this is a psychophysical technique, based on subjective responses, that may not reflect small nerve fibers dysfunctions. The presence of objective signs of small-fiber impairment should be followed by monitoring of possible metabolic and/or immunological and/or paraneoplastic basis, with basal assessment of blood tests [67]. Such tests could be repeated at 1-year follow-up (Fig. 2).

## Limitations

This position statement was based on an expert opinion taking into consideration recent literature. However, we did not perform a systematic review, and considered main studies exploring only the last 10 years in PubMed.

## Conclusions

This shared document has the function of helping clinical neurologists and neurophysiologists in the diagnosis of FM disease although we are still far from understanding mechanisms that could sustain FM clinical picture in single cases. To clarify these mechanisms will definitively improve FM treatment with a precision medicine approach.

The altered pain processing at central level and the frequent involvement of somatic and autonomic small fibers justify the interest and the competence of the neurologists, who may give an important aid to the diagnosis of associated central and peripheral nervous system conditions, as well as novel input to the research about the fundamental causes of the disease. Recent studies on voltage-gated ionic channels [3, 68] showed genetic abnormalities in several patients with FM, similar to those found in patients with neuropathic pain.

The correct diagnostic approach to FM could promote the exclusion of the known causes of small-fiber impairment, and the research toward common genetic factors, useful to promote a more specific therapeutic approach.

## Supplementary Information

Below is the link to the electronic supplementary material.

Supplementary file1 (DOCX 30 KB)
