# Supplementary material for: Clinical criteria and diagnostic assessment of fibromyalgia: position statement of the Italian Society of Neurology-Neuropathic Pain Study Group
Source: Neurol Sci. 2023 May 24;44(7):2561–74. doi: 10.1007/s10072-023-06836-3 (PMC10257633; doi:10.1007/s10072-023-06836-3)
Supplement: Supplementary file 1 — Supplementary file1 (DOCX 30 KB) [file 10072_2023_6836_MOESM1_ESM.docx]

**Fibromyalgia and related neuromuscular diseases.**

Fibromyalgia (FM) is characterized by chronic, widespread musculoskeletal pain, fatigue, sleep disorders and other somatic symptoms such as headaches, abdominal pain, myalgia, paresthesias, muscle contractures, stiffness and muscle weakness. However, many of these symptoms are also complained by patients with different types of neuromuscular diseases. Because of this, for a correct diagnosis of FM and associated conditions, it is necessary to consider any other neuromuscular diseases carefully looking at patient history, neurological exam, electrophysiological studies, weakness degree, characteristics of pain (diffuse or localized), muscle bulk, laboratory tests (for example blood count, C-Reactive Protein, muscle enzymes, serologic tests, rheumatoid factor, antinuclear antibody, extractable nuclear antigen, etc). Muscle imaging (magnetic resonance, ultrasonography, etc.) could be also useful for diagnosis of neuromuscular disorders as well as, in selected cases, a muscle biopsy ^1-2^

As regard as muscle pain, fatigue or related symptoms, several conditions can realize a clinical picture of FM, such as Inflammatory myopathies (Inclusion Body Myositis-IBM, Polymyositis-PM, Dermatomyositis-DM), Myasthenia gravis (MG), Metabolic myopathies, Endocrine myopathies, Toxic myopathies and Mitochondrial myopathies (MM). As regard autonomic involvement with specific attention to gastroenterological symptoms, a differential diagnosis with a neuropathy due to Hereditary Transthyretin Amyloidosis (hATTR) should be considered as well as an acute intermittent porphyria (**see Table 1**)

As mentioned, muscle pain and/or fatigue can be present in IBM, an idiopathic myopathy more frequent in in patients over 50 years old. IBM is characterized by slow progressive painless muscular weakness and muscle atrophy, selective finger flexor weakness but also isolated quadriceps weakness or neck extensor weakness. CK levels are normal or slightly increased (PM/DM show high CK levels); electromyography(EMG) display a typical myopathic pattern and typical features are “rimmed vacuoles” at the muscle biopsy^3^.

In DM/PM, a mild pain is present in a limited number of cases as well as proximal limb muscles weakness and muscle atrophy in the late stages. DM shows specific cutaneous signs (Heliotrope rash, Gottron papules, shawl sign, V sign, mechanic's hands). PM/DM could be associated with cardiomyopathy and/or interstitial lung disease, with, in 20-30 % of patients, presence of antibodies anti-JO1 or anti-SS-A/Ro, anti SS-B/La, anti-Scl-70, ANA^4-5^

MG is mainly characterized by excessive fatigue with a characteristic daily fluctuation. Presence of antibodies as Acetylcholine receptor antibody (anti-AChR) or anti-MuSK and positive single fiber EMG (SFEMG) can help in making MG diagnosis^6-7^.

Toxic myopathies as Statin-Induced Myopathy, often cause myalgia, but other drugs could provoke it such as colchicine, steroids, HIV drugs, macrolides, amiodarone and calcium channel blockers. Statin-Induced Myopathy characterized by diffuse myalgia, proximal muscle weakness, increased CK levels, especially at early stages of therapy^8^. Anti-HMG-CoA reductase antibodies in statin users and anti-SRP antibodies are responsible of rare cases of an Immune-Mediated Necrotizing Myopathy with diffuse muscle pain^9^.

Endocrine myopathies, mainly due to thyroid dysfunction, can be responsible of muscle pain, muscle weakness and fatigue, with CK levels normal or slightly increased^10^.

Between metabolic myopathies, glycogen storage diseases as myophosphorylase deficiency (McArdle Disease or type V glycogenosis) and Pompe Disease (or type II glycogenosis) are characterized by muscle contractures, exercise intolerance, pain and, at later stages, muscle weakness. In McArdle disease, patients frequently report muscle contractures, after even mild physical activity but with a little recovery, these patients typically show the "second-wind" phenomenon, a sudden decrease in heart rate (HR) and an improved exercise tolerance after having restarted the physical exercise. For the final diagnosis it is necessary a muscle biopsy or a molecular genetic analysis ^11^. The adult form of Pompe disease (LOPD) is a slowly progressive proximal myopathy with a following respiratory insufficiency due to a severe diaphragm weakness. CK levels are normal or mildly increased, EMG shows several abnormalities such as bizarre high-frequency discharges, fibrillation potentials and sporadic myotonic discharge mostly at paraspinal muscles. Diagnosis of Pompe Disease requires a Dried Blood Spot (DBS) assay followed, in case of alfa glucosidase reduction (GAA), by a genetic testing ^12-13^.

MM may present with muscle weakness, pain and exercise intolerance that progressively worsens over time. The common histological change, termed “Ragged red fibers (RRF), may disclose a mitochondrial disorder^14^. Mitochondrial Neuro-Gastrointestinal Encephalo-Myopathy (MNGIE) is a rare autosomal recessive disease caused by mutations of the Thymidine Phosphorylase gene and is characterized by severe gastrointestinal symptoms as well as nausea, vomiting, early satiety, abdominal pain, diarrhoea, sub-occlusive episodes, weight loss, cachexia with normal nutritional intake and neurological symptoms such as chronic progressive external ophthalmoplegia (CPEO), ptosis and polyneuropathy. Diagnosis needs to be confirmed by specific genetic tests ^15^.

Hereditary Transthyretin Amyloidosis (hATTR), is a progressive systemic disease with autosomal dominant trait; common phenotypic aspects are fatigue, progressive axonal polyneuropathy, dysautonomia and hypertrophic cardiomyopathy^16^.

Acute intermittent porphyria is an autosomal dominant disease characterized by recurrent attacks of abdominal pain and vomiting, especially precipitated by some drugs such as sulphonamides and anticonvulsant, acute and rapidly progressive sensorimotor polyneuropathy (more or less symmetrical), psychosis (delirium or confusion), convulsions, tachycardia, hypertension. Diagnosis is confirmed by high levels of porphobilinogen and 5-aminolevulinic acid in the urines and by genetic studies^17^.

**Table 1**

| **Disease** | **Common aspects to FM** | **Other clinical features** | **Diagnosis** |
| --- | --- | --- | --- |
| **Inclusion Body Myositis (IBM) ^3^** | Muscle pain and/or fatigue | - Muscle weakness and atrophy - selective finger flexor weakness or isolated quadriceps weakness or neck extensor weakness are characteristics pattern of involvement. | **CK levels** normal or slightly increased  **EMG**:myopathic pattern  **Muscle biopsy**: “ rimmed vacuoles”  **Genetic test** |
| **Polymyositis (PM), Dermatomyositis (DM) ^4-5^** | Mild muscle pain  (in limited number of patients), fatigue and muscle weakness | - Proximal limb muscles weakness and muscle atrophy - specific cutaneous signs - cardiomyopathy and/or interstitial lung disease, | **CK levels** increased  **EMG**:myopathic pattern  **Serologic test**: ant-JO1,etc.  **Muscle Biopsy** |
| **Myasthenia gravis (MG) ^6-7^** | Fatigue and muscle weakness | - Excessive fatigue with a characteristic daily fluctuation. - Ptosis, dysphagia,dysphonia | **SFEMG:** mean jitter value increased  **Serologic tests:** anti-AChR, anti-MuSK, etc. |
| **Toxic myopathies^8-9^** | Myalgia, hyperCKemia | - No typical sign | Improvement with withdrawal/ changing drug |
| **Endocrine myopathies^10^** | Muscle pain, muscle weakness and fatigue | - Typical sign of endocrine disorders as hypothyroidism (exophthalmos, myxedema, etc) | **Typical endocrinological aspects**  **CK levels** normal or slightly increased |
| **McArdle Disease^11^** | Muscle pain, weakness, fatigue | - Contractures, “second-wind phenomenon”, episodes of myoglobinuria, etc.^12^ | **CK levels** highly increased  **Muscle Biopsy**  **Genetic Test** |
| **Adult Pompe Disease (LOPD) ^12-13^** | Muscle pain, muscle weakness, fatigue | - **Proximal muscle** weakness, respiratory insufficiency | **CK levels** normal or slightly increased  **DBS**  **Genetic Test** |
| **Mitochondrial myopathies (MM) ^14-15^** | Muscle weakness, pain, and exercise intolerance | - Progressive External Ophthalmoplegia (PEO) - Muscle weakness - Multisystem disorder | **Genetic Test** |
| **Hereditary Transthyretin Amyloidosis^16^** | Gastrointestinal symptoms, autonomic involvement | - Polyneuropathy, - Cardiomyiopathy,Dysautonomia - Vitreal opacities, etc. | **Genetic Test** |
| **Acute intermittent porphyria^17^** | Abdominal pain, vomiting, nausea | - Rapidly progressive sensorimotor polyneuropathy - Psychosis - Convulsions | High levels of **porphobilinogen and 5-aminolevulinic** acid in the urines  **Genetic test** |

**References**

1. Mastaglia FL, Garlepp MJ, Phillips BA et al. (2003) Inflammatory myopathies: clinical, diagnostic and therapeutic aspects. MuscleNerve 27: 407–425
2. Filosto M, Tonin P, Vattemi G et al. (2007) The role of muscle biopsy in investigating isolated muscle pain. Neurology 68: 181– 186
3. Dimachkie, Mazen M., and Richard J. Barohn. (2014) "Inclusion body myositis." *Neurologic clinics* 32.3: 629-646..
4. Lundberg, Ingrid E., et al.(2016) "Diagnosis and classification of idiopathic inflammatory myopathies." *Journal of internal medicine* 280.1 : 39-51.
5. Sasaki, Hirokazu, and Hitoshi Kohsaka. (2018) "Current diagnosis and treatment of polymyositis and dermatomyositis." Modern rheumatology 28.6: 913-921.
6. Patel P, Pobre T. (2022) Electrodiagnostic Evaluation Of Neuromuscular Junction Disorder. 2022 Sep 5. In: StatPearls [Internet]. Treasure Island (FL): StatPearls Publishing; 2022 Jan–. PMID: 32965973.
7. Rodolico, Carmelo, et al. (2020) "MuSK-associated myasthenia gravis: clinical features and management." *Frontiers in Neurology* 11 : 660.
8. Sieb, Joern P., and Thomas Gillessen. (2003) "Iatrogenic and toxic myopathies." Muscle & Nerve: Official Journal of the American Association of Electrodiagnostic Medicine 27.2: 142-156.
9. Pinal-Fernandez, Iago, Maria Casal-Dominguez, and Andrew L. Mammen.(2018) "Immune-mediated necrotizing myopathy." Current Rheumatology Reports 20 : 1-10.
10. Lochmüller H, Reimers CD, Fischer P et al.(1993) Exercise-induced myalgia in hypothyroidism. Clin Invest 71: 999–1001. doi:10.1007/bf00180031
11. Quinlivan, Ros, et al. (2010) "McArdle disease: a clinical review." *Journal of Neurology, Neurosurgery & Psychiatry* 81.11 : 1182-1188.
12. Toscano A, Rodolico C, Musumeci O. [Multisystem late onset Pompe disease (LOPD): an update on clinical aspects.](https://www.ncbi.nlm.nih.gov/pubmed/31392196) (2019) Ann Transl Med. 2019 Jul;7(13):284. doi: 10.21037/atm.2019.07.24. Review. PMID: 31392196.
13. Gesquière‐Dando, Aude, et al. (2015) "Fibromyalgia‐like symptoms associated with irritable bowel syndrome: A challenging diagnosis of late‐onset Pompe disease." Muscle & Nerve 52.2 : 300-304.
14. Abdullah, Mishal, et al.(2012) "Mitochondrial myopathy presenting as fibromyalgia: a case report." *Journal of Medical Case Reports* 6 : 1-3.
15. Hirano, Michio, et al. (2021) "Mitochondrial neurogastrointestinalencephalomyopathy (MNGIE): Position paper on diagnosis, prognosis, and treatment by the MNGIE International Network." Journal of inherited metabolic disease 44.2 : 376-387.
16. Adams, David, et al. (2021) "Expert consensus recommendations to improve diagnosis of ATTR amyloidosis with polyneuropathy." *Journal of neurology* 268: 2109-2122.
17. Cardenas, John Lidemberto, and Carlos Guerrero. (2018) "Acute intermittent porphyria: general aspects with focus on pain." Current medical research and opinion 34.7 : 1309-1315.
